# Mucormycosis in the Jaw: A Report of 2 Cases and Literature Review

**DOI:** 10.3290/j.ohpd.a45522

**Published:** 2020-11-20

**Authors:** Eun-Jung Kwak, Dong-Jin Kim, Woong Nam, Wonse Park

**Affiliations:** a Clinical Professor, National Dental Care Center for Persons with Special Needs, Seoul National University Dental Hospital, Seoul, Korea. Study concept and design, drafted and critically revised manuscript for important intellectual content, contributed substantially to discussion, final approval of the version to be published, contributed equally to this work.; b Resident, Department of Advanced General Dentistry, College of Dentistry, Yonsei University, Seoul, Korea. Data acquisition, contributed substantially to discussion, final approval of the version to be published, contributed equally to this work.; c Professor, Department of Oral and Maxillofacial Surgery, College of Dentistry, Yonsei University, Seoul, Korea. Contributed substantially to discussion, final approval of the version to be published.; d Professor, Department of Advanced General Dentistry, College of Dentistry, Yonsei University, Seoul, Korea. Study concept, contributed substantially to discussion, final approval of the version to be published.

**Keywords:** jaw, mucormycosis, necrosis, zygomycosis

## Abstract

Mucormycosis is a rare fungal infection with high morbidity and mortality and a very poor prognosis. However, aggressive medical and surgical management can result in survival rates exceeding 80%. The most common sites involved in mucormycosis infection are the sinus, lung, skin and soft tissues, gastrointestinal system, central nervous system, and rarely the mandible. Systemic risk factors for mucormycosis are diabetes mellitus (DM), neutropenia, corticosteroid use, hematologic malignancies, organ transplantation, metabolic acidosis, deferoxamine use, and advanced age. Local risk factors are a history of trauma, burns, surgery. We report on two patients with mucormycosis of the jaw. While one case presented as mucormycois involving the maxillary sinus in a patient with uncontrolled DM, the other was a rare case of mandibular mucormycosis in a patient with acute myeloid leukemia.

Mucormycosis is an infection by fungi of the Mucorales order,^3^ characterised by vessel invasion and tissue necrosis that occurs mainly in immunocompromised patients. Several immunosuppressive conditions, such as poorly controlled diabetes mellitus (DM), hematologic malignancy, solid-organ transplantation, chronic renal failure, nutritional deficiency, severe burns, and trauma act as predisposing factors for opportunistic fungal infections.^2^ The first case of mucormycosis in humans was reported in 1885 by Paltauf, and a case of rhino-orbital cerebral mucormycosis was reported by Gregory et al in 1943.^9^ Mucormycosis presents with various clinical features and mainly involves rhinocerebral lesions, the skin, lung, central nervous system, and gastrointestinal tract. Mucormycosis can secondarily extend from the primary infection site via hematogenous dissemination,^19^ and in uncommon cases, it infects the kidney, heart, and oral cavity.

The most frequently involved site in oral and maxillofacial regions is the maxillary sinus, where it presents with tissue necrosis. Without appropriate treatment it can extend to the orbit and brain, which has a poor prognosis. Mucormycosis can also occur in the maxillary alveolar ridges, lips, tongue, and mandible,^11^ but mandibular mucormycosis is extremely rare. Mucormycosis has a very poor prognosis, but appropriate treatment that includes active antifungal therapy and aggressive surgical debridement can increase the survival rate to 80%.^25^ However, even after successful treatment, mucormycosis can become dormant and then recur during chemotherapy or neutropenia.^23^

This report of two rare cases of oral mucormycosis involves one originating from the maxillary sinus and infiltrating the palate in a patient with uncontrolled DM, and the other of mucormycosis infection in the gingiva and alveolar bone of the mandible in a patient with acute myeloid leukemia.

## Case Reports

### Case 1

A 44-year-old man was referred to the Department of Advanced General Dentistry at Yonsei University College of Dentistry from the Department of Infection Medicine, Severance Hospital, to address mobility of the left maxillary incisor. Clinical and radiographic examinations showed grade-2 mobility of the maxillary left central incisor, lateral incisor, and canine, and enlargement of periodontal ligament space. The patient had been admitted to the Department of Otorhinolaryngology, Severance Hospital, after receiving endoscopic sinus surgery and Caldwell-Luc surgery for sinus fungal infections before the present referral. The initial pathologic diagnosis was invasive aspergillosis, and the patient was transferred to the Department of Infection Medicine for antifungal therapy and was maintained on intravenous voriconazole. This patient also had systemic diseases: hypertension, hyperlipidemia, hypothyroidism, and uncontrolled DM (glucose: 377 mg/dl, HbA1c: 14.4%).

**Fig 1:**
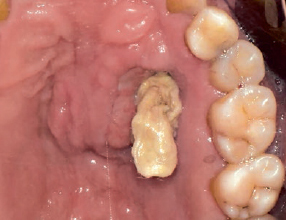
Clinical photograph showing swelling of the left hard palate and yellowish flabby tissue.

**Fig 2:**
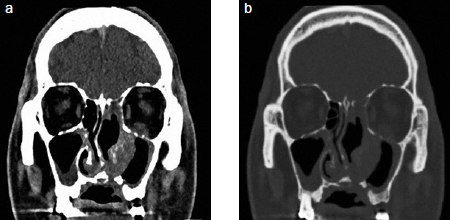
Computed tomography view showing bony erosion of the left hard palate and maxillary sinus walls. a: Soft-tissue window view; b: bone window view.

We also observed swelling and yellowish-white elongated tissue on the left hard palate (Fig 1). The patient mentioned that it did not subside on the left hard-palate tissue after eating hot food, but we diagnosed it as being associated with the existing sinus fungal infection related to the medical history of the patient. Computed tomography (CT) and wide resection through cooperation with an oral maxillofacial surgeon was necessary. Thus, we recommended prompt intervention to the Department of Infection Medicine. CT revealed bony erosion of the left hard palate and maxillary sinus walls (Fig 2).

**Fig 3:**
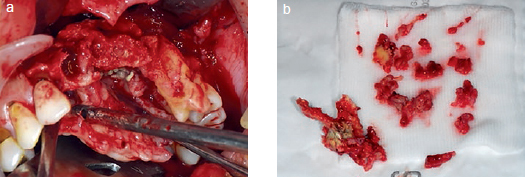
a: Partial maxillectomy was performed; b: specimen.

**Fig 4:**
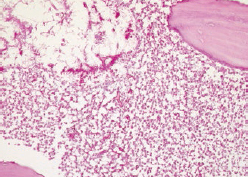
Histopathologic findings (PAS stain, 200X).

**Fig 5:**
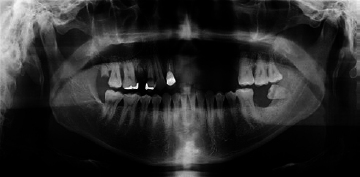
Postoperative panoramic radiograph (case 1).

Partial maxillectomy with extraction of the left lateral incisor, canine, and first and second premolars was then performed (Fig 3). The specimens were sent to the Department of Oral Pathology, and the histopathological results confirmed the presence of mucormycosis, which manifests as a broad and irregular form of nonseptate branching hyphae (Fig 4). Intravenous voriconazole administered following the initial pathologic diagnosis was changed to intravenous AmBisome (amphotericin B) in accordance with the final pathologic diagnosis. Its rapidly spreading behaviour had resulted in it extending to the orbital and cavernous sinus in this patient. However, the application of active and appropriate antifungal therapy improved the patient’s condition, and at the last study follow-up, he was undergoing prosthetic treatment of the maxillary region (Fig 5).

### Case 2

A 61-year-old man diagnosed with acute myeloid leukemia (AML) was admitted to the Department of Hematology, Severance Hospital, for chemotherapy. This patient was referred to the Department of Advanced General Dentistry due to swelling, soreness, and a heat sensation in the left cheek area. Other systemic diseases of this patient were hypertension and poorly controlled DM (HbA1c: 8.0%). The patient had not been to a dentist for 20 years. Clinical and radiographic examinations showed grade-3 mobility of the mandibular left first and second molars. Root caries were also present.

**Fig 6:**
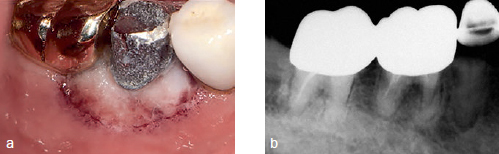
Clinical photograph and periapical view. a: Grey and fibrotic gingiva with well-delineated sloughing border are observed; b: alveolar bone resorption is shown.

**Fig 7:**
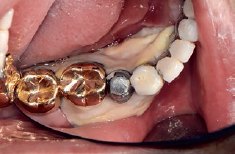
Swelling of right mandibular gingiva, with whitish necrotic tissue evident.

**Fig 8:**
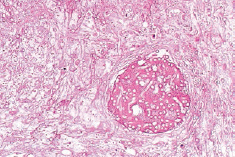
Histopathologic findings (H&E stain). Vascular invasion and fungal hyphae are observed (200X).

The patient’s teeth were extracted and chemotherapy was resumed for AML. One month after the extraction, the patient was referred again for swelling and pain in the gingiva of the right mandibular second premolar and the first molar region. Clinical and radiographic examinations showed fibrous gingiva and bone loss under the mandibular second premolar and first molar. An incisional biopsy was planned (Fig 6), but at the time of referral the patient had a WBC count of 240/µl, hemoglobin level of 9.1 g/dl, hematocrit of 25.7%, and platelet count of 23,000/µl, meaning that a biopsy procedure was not possible. After 15 days the blood counts had improved to within the normal ranges and so the biopsy was performed. The gingiva in the posterior part of the right mandible was necrotic, and the necrosis extended to the anterior part (Fig 7). The biopsy specimen was sent to the Department of Oral Pathology and was confirmed as mucormycosis. Intravenous AmBisome (amphotericin B) 200~320 mg/day was administered. In the pathology specimen, a mycelium with an obtuse angle without a septum was observed in the blood vessels (Fig 8).

**Fig 9:**
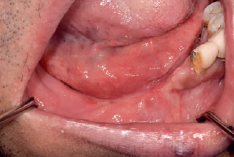
Postoperative intraoral clinical photograph.

At 5 days after the biopsy, the patient underwent extraction of the mandibular right first premolar, second premolar, first molar, and second molar followed by sequestrectomy. After the diagnosis of mucormycosis, intravenous amphotericin B was administered for 4 weeks to prevent recurrence. However, amphotericin B induced a shock response and so the medication was changed to Noxafil (posaconazole) 300 mg/day for about 35 days. Prophylactic therapy and chlorhexidine rinse were also applied periodically to prevent further infections. At 1 month after the operation, there were no signs of mucormycosis or necrotic tissue in the infected area of the patient, and no sign of additional infection was observed (Fig 9).

**Fig 10:**
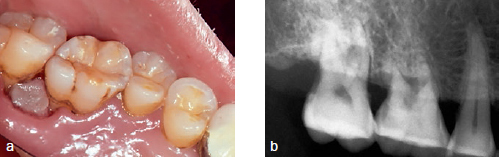
a: Swelling of right maxillary gingiva, whitish necrotic tissue observed; b: no resorption of the alveolar bone is evident.

The patient was referred again at 2 months after the operation for right maxillary gum pain and oedema. A clinical examination showed a whitish lesion and gingival recession under the maxillary right first and second molars, and mucormycosis was confirmed by the subsequent biopsy (Fig 10). There was no loss of alveolar bone in the infected area radiographically; thus, it was considered that mucormycosis was limited to the gingiva. Periodic scaling and chlorhexidine rinse were therefore performed, but the response was poor. Hence, both maxillary right first and second molars were extracted. The patient was subsequently monitored for recurrence of mucormycosis in periodic visits.

## Discussion

Mucormycosis is a rare but life-threatening infectious disease with a high mortality rate.^5^ The risk factors for mucormycosis are poorly controlled DM, neutropenia, hematopoietic malignancy (leukemia, lymphoma, and multiple myeloma), solid-organ transplantation, multiple traumas, and use of corticosteroids.^16^ Reports of mucormycosis have recently increased worldwide, especially in patients with DM or malignant tumors.^20^ Mucormycosis presents in various forms: rhino-orbito-cerebral, pulmonary, gastrointestinal, cutaneous, and disseminated.^14^ The oral form of mucormycosis is relatively rare,^19^ The main affected area in the oral and maxillofacial region is the maxillary sinus, and it can present with invasion and necrosis of the palate. Without appropriate treatment, it will expand to the orbit and brain, which has a very poor prognosis.^22^ Besides the maxillary sinus, mucormycosis in the alveolar bone of the maxilla, lip, tongue, and mandible has been reported. However, cases involving the mandible are very rare.^2^,^4^,^8^,^11^,^23^ The first case of mandible-affected mucormycosis was reported by Eisenberg et al,^13^ and to date, 12 such cases have been reported, as presented in Table 1.

**Table 1:** Reported cases of mucormycosis involving the mandible

Authors and year	Age, years	Sex	Predisposing diseases or risk factors
Eisenberg et al (1977)^8^	28	Female	Corticosteroid use, acute renal failure, metabolic acidosis, hyperglycemia
Brown and Finn (1986)^4^	57	Male	Acute renal failure, DM
Jones et al (1993)^11^	43	Male	Acute myeloid leukemia, acute renal failure
Salisbury et al (1997)^23^	60	Male	Acute myeloid leukemia, prostate cancer
Lador et al (2006)^13^	42	Female	Acute lymphoblastic leukemia
Dogan et al (2007)^7^	7	Male	Acute myeloid leukemia
Antonetti et al (2009)^1^	10	Male	Burns
Ojeda-Uribe et al (2010)^17^	55	Female	Acute myeloid leukemia
Aras et al (2012)^2^	15	Male	Acute myeloid leukemia
Aras et al (2012)^2^	6	Male	Neuroblastoma
Oswal et al (2012)^18^	68	Female	DM, hypertension
Magister et al (2015)^15^	70	Female	Acute myeloid leukemia
Our case 2	61	Male	Acute myeloid leukemia

Despite the relatively favourable prognosis of mucormycosis with adequate surgical resection and antifungal therapy, it is difficult to define treatment of the case of mucormycosis in the mandible. Due to suboptimal blood supply, it is easily transmitted to the necrotic gingiva and bone in spite of the early diagnosis; appropriate surgery as well as repeated dormancy and recurrence will prolong the time to complete healing. Also, mucormycosis in the mandible is more likely to be associated with hematologic malignancy than with diabetes mellitus, corticosteroid use, and trauma. As shown in Table 1, the patient had acute leukemia in 8 out of 13 cases, including 12 reported cases and 1 case in this case report. Especially acute leukemia patients who are undergoing chemotherapy or bone marrow transplantation suffer from neutropenia. Neutrophils have an important role in host defense system against fungal colonisation. In patients with leukemia, bone marrow fails to produce healthy red or white blood cells or platelets. Moreover, myelosuppression and neutropenia occur during remission and induction before and after chemotherapy. These situations render patients relatively vulnerable to fungal infections and make them prone to opportunistic infections.^23^ Close follow-up is necessary, because it reveals the process of dormancy and reappearance in the treatment of mucormycosis. In case 2, the unfavourable condition of acute myeloid leukemia combined with uncontrolled diabetes and age factor led to invasive mandible mucormycosis, despite close follow-up.

The diverse clinical presentations of mucormycosis make early diagnosis difficult. However, patients with risk factors and deteriorating progress despite taking empirical antibiotics must be examined for the possibility of mucormycosis infection. Unlike aspergillosis, it is difficult to diagnose mucormycosis using serological and imaging techniques, so that histopathological examination and culturing are necessary. The histopathological hallmarks of mucormycosis are broad and irregularly shaped nonseptate branching hyphae 10~50 μm in size with vessel invasion by thrombosis and tissue necrosis.^3^,^10^,^24^ Successful early diagnoses were recently reported based on C-reactive protein and the polymerase chain reaction, but further investigations are essential.^21^

The principles of mucormycosis treatment are early diagnosis, removal of risk factors, debridement of infected tissue, surgical resection, and effective antifungal therapy.^12^ Characteristics of mucormycosis such as vessel invasion with thrombosis and tissue necrosis impede the ability of antifungal agents to reach an infectious region, and so aggressive surgical resection and systemic antifungal therapy for removing the infection source is necessary. A lipid amphotericin B formulation (5~10 mg/kg/day) is the first-choice antifungal treatment agent, while amphotericin B deoxycholate is no longer recommended due to renal toxicity. If treatment failure or drug side effects occur when administering liposomal amphotericin B, posaconazole or liposomal amphotericin B combined with posaconazole is recommended.^6^ Because a shock event occurred in case 2 when liposomal amphotericin B was prescribed for 4 weeks, the medication was changed to posaconazole. Mucormycosis is a life-threatening infectious disease whose mortality rate depends on the invasion area.^20^ The survival rate could be increased by early diagnosis, appropriate antifungal therapy, and management of predisposing factors.^16^

## Conclusion

The two patients presented here represent very rare cases of mucormycosis originating from the maxillary sinus with invasion to the palate and mandible. Because no accurate guideline exists about surgical resection due to a paucity of reported cases, the applied treatment might depend on the experience of the clinician. The present case series demonstrates the importance of early detection, diagnosis, and appropriate treatment of mucormycosis.
